# The association between changes in lifestyle behaviors and the incidence of chronic kidney disease (CKD) in middle-aged and older men

**DOI:** 10.1016/j.je.2016.08.013

**Published:** 2017-04-03

**Authors:** Ryoma Michishita, Takuro Matsuda, Shotaro Kawakami, Satoshi Tanaka, Akira Kiyonaga, Hiroaki Tanaka, Natsumi Morito, Yasuki Higaki

**Affiliations:** a Department of Health Development, Institute of Industrial Ecological Sciences, University of Occupational and Environmental Health, Kitakyushu, Japan; b The Fukuoka University Institute for Physical Activity, Fukuoka, Japan; c Department of Rehabilitation, Fukuoka University Hospital, Fukuoka, Japan; d Laboratory of Exercise Physiology, Faculty of Health and Sports Science, Fukuoka University, Fukuoka, Japan; e Fukuoka University Health Care Center, Fukuoka, Japan; f Department of Cardiology, Fukuoka University School of Medicine, Fukuoka, Japan

**Keywords:** Incidence of CKD, The changes in lifestyle behaviors, Habitual moderate exercise, Late-night dinner, Bedtime snacking

## Abstract

**Background:**

This study was designed to evaluate whether changes in lifestyle behaviors are correlated with the incidence of chronic kidney disease (CKD).

**Methods:**

The subjects consisted of 316 men without a history of cardiovascular disease, stroke, or renal dysfunction or dialysis treatment. The following lifestyle behaviors were evaluated using a standardized self-administered questionnaire: habitual moderate exercise, daily physical activity, walking speed, eating speed, late-night dinner, bedtime snacking, skipping breakfast, and drinking and smoking habits. The subjects were divided into four categories according to the change in each lifestyle behavior from baseline to the end of follow-up (healthy–healthy, unhealthy–healthy, healthy–unhealthy and unhealthy–unhealthy).

**Results:**

A multivariate analysis showed that, with respect to habitual moderate exercise and late-night dinner, maintaining an unhealthy lifestyle resulted in a significantly higher odds ratio (OR) for the incidence of CKD than maintaining a lifestyle (OR 8.94; 95% confidence interval [CI], 1.10–15.40 for habitual moderate exercise and OR 4.00; 95% CI, 1.38–11.57 for late-night dinner). In addition, with respect to bedtime snacking, the change from a healthy to an unhealthy lifestyle and maintaining an unhealthy lifestyle resulted in significantly higher OR for incidence of CKD than maintaining a healthy lifestyle (OR 4.44; 95% CI, 1.05–13.93 for healthy–unhealthy group and OR 11.02; 95% CI, 2.83–26.69 for unhealthy–unhealthy group).

**Conclusions:**

The results of the present study suggest that the lack of habitual moderate exercise, late-night dinner, and bedtime snacking may increase the risk of CKD.

## Highlights

• The lack of exercise habit is associated with the incidence of CKD.
• Late-night dinner and bedtime snacking increase the risk of CKD.
• Early lifestyle intervention is necessary to prevent CKD incidence.

## Introduction

The number of patients with end-stage renal disease (ESRD) in Japan is continuously increasing.^1^ Chronic kidney disease (CKD) has been related to the development of ESRD and cardiovascular disease (CVD) morbidity and mortality.^2^, ^3^ The large number of ESRD patients at present is believed to be associated with the increase in number of patients with CKD. It is well known that aging, hypertension, diabetes mellitus, and metabolic syndrome are risk factors for CKD.^4^, ^5^, ^6^

In addition to the above factors, the incidence of CKD is also closely related to unhealthy lifestyle behaviors, such as smoking, heavy alcohol intake, obesity, lack of exercise habit, and unhealthy eating styles.^7^, ^8^, ^9^, ^10^, ^11^, ^12^ In our previous cross-sectional and longitudinal studies,^13^, ^14^ we observed that reduced incidence of CKD correlated with an increase in the number of healthy lifestyle behaviors. Adherence to healthy lifestyle behaviors is therefore thought to be important to prevent the incidence of CKD. However, no study has that evaluated the effect of lifestyle behaviors changes on preventing the onset of CKD through long-term follow-up.

The purpose of lifestyle modification in improving CKD is not only to increase renal function, but also to prevent the development of ESRD and CVD. Clarification of the association between the changes in lifestyle behaviors and the incidence of CKD may contribute to understanding the importance of lifestyle modification in CVD and ESRD prevention. We therefore hypothesized that maintaining or improving healthy lifestyle behaviors may help reduce the development of CKD, as the accumulation of healthy lifestyle behaviors has been demonstrated to be associated with prevention of the development of CKD.^13^, ^14^ This study retrospectively investigated the influence of changes in lifestyle behaviors on the incidence of CKD in middle-aged and older men.

## Methods

### Subjects

**Fig. 1:**
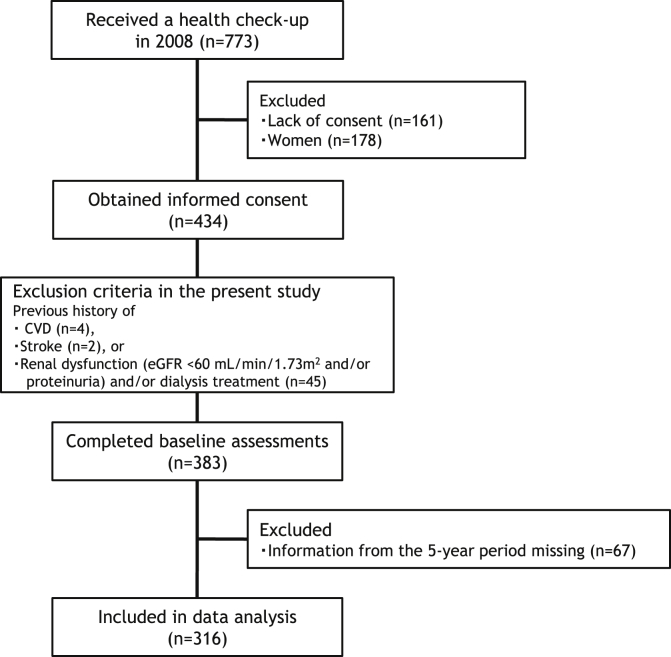
A flow-chart of the participants included in the study. CVD, cardiovascular disease; eGFR, estimated glomerular filtration rate.

A total of 773 middle-aged and older adults received their periodic health check-up at a health care center in Fukuoka University in 2008. The flow of participants through the study is shown in Fig. 1. The method for this health check-up has already been described in a previous study.^14^ Of the 434 subjects who provided informed consent, 178 women were excluded to remove the influence of gender. Subjects with a previous history of CVD (n = 4), stroke (n = 2), renal dysfunction (estimated glomerular filtration rate [eGFR] using the Japanese coefficient-modified CKD-Epidemiology Collaboration [EPI] equation <60 mL/min/1.73 m^2^, or proteinuria, or both^15^), and/or dialysis treatment (n = 45), were also excluded from the analysis. A total of 316 men (mean age: 52.5 [standard deviation {SD}, 6.7] years; mean body mass index [BMI]: 23.3 [SD, 2.7] kg/m^2^; mean serum creatinine [Cr]: 0.84 [SD, 0.09] mg/dL; and mean eGFR: 81.2 [SD, 6.1] mL/min/1.73 m^2^) with no missing information during the follow-up were deemed eligible for the present study.

All of the subjects gave their informed consent for participation after agreeing with the purpose, methods, and significance of the study. The study conforms to the Declaration of Helsinki and was approved by the Ethics Committee of Fukuoka University (No. 11-08-01).

### Blood sampling, blood pressure, and anthropometry measurements

Blood samples were collected early in the morning via venipuncture from an antecubital vein after at least 12 h of fasting. The blood samples were analyzed by Special Reference Laboratories (SRL Inc., Tokyo, Japan). The serum Cr, high-density lipoprotein cholesterol (HDL-C) and low-density lipoprotein cholesterol (LDL-C) levels were measured using the direct method. The triglyceride level was measured using the enzyme method. The plasma glucose level was measured using an ultraviolet/hexokinase method and hemoglobin A_1_c (HbA_1_c) was measured via high-performance liquid chromatography. HbA_1_c is presented as the National Glycohemoglobin Standardization Program (NGSP) value, which was calculated using the conversion equation for HbA_1_c derived from the Japan Diabetes Society (JDS): HbA_1_c (NGSP value; %) = 1.02 × JDS value (%) + 0.25%.^16^

The eGFR was calculated using the CKD-EPI equations modified by a Japanese coefficient: eGFR (mL/min/1.73 m^2^) = Cr ≤ 0.9 mg/dL, 141 × (Cr/0.9)^−0.411^ × 0.993^age^ × 0.813; Cr > 0.9 mg/dL, 141 × (Cr/0.9)^−1.209^ × 0.993^age^ × 0.813.^17^ The eGFR is a more accurate measure of the renal function than serum Cr^18^ and identifies patients who have mild renal impairment despite having normal or nearly normal Cr levels. Furthermore, the eGFR is a strong predictor of cardiovascular events and is more useful for this purpose than serum Cr.^19^, ^20^ A urinalysis for assessment of the proteinuria and hematuria were performed using a dipstick, and the urine test results were classified as (−), (±), (1+), (2+), or (3+).^21^ In this study, CKD was defined according to definition of the Japanese Society of Nephrology (eGFR < 60 mL/min/1.73 m^2^, proteinuria positive [1+ or greater], or both).^15^ The breakdown of subjects' CKD grade at baseline^16^ was as follows: G1 (eGFR ≥ 90 mL/min/1.73 m^2^), n = 21 (6.6%); and G2 (eGFR 60–89 mL/min/1.73 m^2^), n = 295 (93.4%).

Blood pressure was measured in the right arm with the subject sitting in a chair, after more than 5 min of rest, and was expressed as an average of duplicate measurements. The height and body weight were measured, and the BMI was calculated as the ratio of body weight (kg) to height squared (m^2^). The waist circumference was measured at the level of the umbilicus.

### Assessment of lifestyle behaviors

The subjects' lifestyle behaviors regarding exercise, physical activity, eating style, and drinking and smoking habits were evaluated using the standardized self-administered questionnaire of the National Health Promotion Program.^22^, ^23^ The contents for this questionnaire were described in a previous study.^13^, ^14^ The subjects' lifestyle behaviors regarding physical activity, exercise, eating style, and drinking and smoking habits were determined based on their responses to the following questionnaire items: habitual moderate exercise, ≥30 min at a time and ≥2 times per week (yes or no); physical activity, equal to walking at least 1 h per day (yes or no); walking speed, compared with people of the same sex and age group (fast or slow); eating speed, compared with others (fast or slow); late-night dinner, ≥3 times per week (yes or no); bedtime snacking, ≥3 times per week (yes or no); and skipping breakfast, ≥3 times per week (yes or no). The subjects' drinking and smoking habits were assessed using the following questionnaire items (with “yes” or “no” responses): drinking habit (not drinking everyday); and smoking habit (recently not smoking). In this study, the lack of exercise habit (less than 30 min at a time and 2 times per week), low-physical activity level (less than 1 h per day), slow-walking speed (compared with people of the same sex and age group), high eating speed (compared with others), late-night dinner (more than 3 times per week), bedtime snacking (more than 3 times per week), and skipping breakfast (more than 3 times per week) were defined as unhealthy lifestyle behaviors. The subjects were divided into four categories according to the change in each of the above lifestyle behaviors from baseline to end-point year, as follows: healthy–healthy, unhealthy–healthy, healthy–unhealthy, and unhealthy–unhealthy. Because no subjects stared smoking again after they quit smoking, subjects' smoking habit was classified into only three categories: healthy–healthy, unhealthy–healthy, and unhealthy–unhealthy.

### Statistical analysis

In this study, the subjects' end-point of follow-up was the onset year of CKD, and the biochemical analysis, blood pressure, and anthropometry measurements and assessment of lifestyle behaviors were conducted at baseline (2008) and the end-point year (maximal 5 years [until 2013]). As a result, the present study only analyzed data from subjects who had undergone a periodic health check-up at baseline and the end-point year. The data were expressed as the mean value with the SD. Statistical analyses were performed using the StatView 5.0 software package (SAS Institute, Cary, NC, USA). In this study, the subjects' lifestyle behaviors were expressed as categorical variables, while other coronary risk factors were shown as continuous variables. The inter-group comparisons were performed using Welch's t-test for continuous variables and the chi-square test for categorical variables. The differences in the eGFR and other coronary risk factors at baseline and after follow-up among the changes in lifestyle behaviors were determined using a two-way repeated-measures analysis of variance and Tukey–Kramer method for the follow-up and groups × time interactions. Comparisons of the data at the baseline and after the follow-up were performed using a Wilcoxon signed-ranks test for continuous variables. Pearson's simple regression and the stepwise multivariate regression analyses were performed in order to determine the associations of the change in eGFR with the changes in other coronary risk factors. A multiple logistic regression analysis was performed to investigate the relationships between the changes in lifestyle behaviors and the incidence of CKD. In this multiple logistic regression analysis, age, BMI, waist circumference, SBP, DBP, LDL-C, HbA_1_c, and eGFR levels at baseline were entered as adjusted factors. A probability value <0.05 was considered to be statistically significant.

## Results

After the follow-up, incident CKD (eGFR < 60 mL/min/1.73 m^2^ and/or proteinuria) was observed in 21 subjects (6.6%). The mean follow-up period was 4.9 (SD, 0.6) years. The breakdown of the subjects by CKD grade^15^ at baseline was as follows: G1 (eGFR ≥ 90 mL/min/1.73 m^2^), n = 20 (6.3%); G2 (eGFR 60–89 mL/min/1.73 m^2^), n = 296 (93.7%), while that after the follow-up was as follows: G1, n = 9 (2.8%); G2, n = 286 (90.5%); and G3a (eGFR 45–59 mL/min/1.73 m^2^), n = 21 (6.6%; including 2 with proteinuria and 2 with microalbuminuria [urinary proteinuria (±)]). In this study, hematuria (1+ or greater) was not observed. Table 1 compares the baseline characteristics in subject who did and did not develop CKD. The serum Cr, systolic blood pressure, and HbA_1_c level were significantly higher (all p < 0.05) and eGFR was significantly lower (p = 0.0001) in the CKD group than in the non-CKD group. No significant differences between these groups were noted in the classifications of CKD grade and other coronary risk factors.

**Table 1:** The baseline characteristics in subjects with and without the development of CKD.

	All (n = 316)	Developed CKD (n = 21)	Did not develop CKD (n = 295)	p value
eGFR, mL/min/1.73m^2^	81.2 (6.1)	76.1 (5.8)	81.6 (6.0)	<0.0001
Classifications of CKD grade				
G1, eGFR ≥ 90 mL/min/1.73 m^2^, n (%)	20 (6.3)	0 (0)	20 (6.8)	0.218
G2, eGFR 60–89 mL/min/1.73 m^2^, n (%)	296 (93.7)	21 (100)	275 (93.2)	
Serum creatinine, mg/dL	0.84 (0.09)	0.92 (0.08)	0.83 (0.09)	<0.0001
Age, years	52.5 (6.7)	52.0 (6.4)	52.5 (6.8)	0.741
Body weight, kg	67.4 (9.2)	67.0 (10.9)	67.4 (9.1)	0.828
BMI, kg/m^2^	23.3 (2.7)	23.5 (3.1)	23.3 (2.7)	0.795
Waist circumference, cm	83.4 (7.4)	84.8 (8.1)	83.3 (7.4)	0.372
SBP, mm Hg	126.4 (14.3)	133.4 (9.1)	125.9 (14.5)	0.020
DBP, mm Hg	82.2 (10.1)	85.5 (8.9)	82.0 (10.1)	0.125
LDL-C, mg/dL	118.6 (27.0)	111.7 (29.0)	119.1 (26.9)	0.226
HDL-C, mg/dL	58.1 (13.6)	57.0 (12.7)	58.2 (13.6)	0.686
Triglyceride, mg/dL	114.8 (77.0)	138.5 (126.2)	116.7 (78.0)	0.114
Fasting glucose, mg/dL	99.9 (16.4)	99.3 (18.3)	99.9 (16.3)	0.873
HbA_1_c, NGSP %, mean (SD)	5.6 (0.6)	5.8 (0.4)	5.6 (0.6)	0.045
Anti-hypertensive drugs, yes/no, n (%)	44 (13.9)/272 (86.1)	5 (23.8)/23 (76.2)	39 (13.2)/255 (86.8)	0.176
Anti-hyperlipidemic agents, yes/no, n (%)	26 (8.2)/290 (91.8)	4 (19.0)/17 (80.0)	22 (7.5)/273 (92.5)	0.062
Hypoglycemic drugs, yes/no, n (%)	10 (3.2)/306 (96.8)	1 (4.8)/20 (95.2)	9 (3.1)/286 (96.9)	0.665

CKD, chronic kidney disease; eGFR, estimated-glomerular filtration rate; BMI, body mass index; SBP, systolic blood pressure; DBP, diastolic blood pressure; LDL-C, low-density lipoprotein cholesterol; HDL-C, high-density lipoprotein cholesterol; HbA_1_c, hemoglobin A_1_c; NGSP, national glycohemoglobin standardization program.

Data are expressed as mean (standard deviation) or number of subjects (%).

The classifications of CKD grade was defined according to definition of the Japanese Society of Nephrology.^15^

Table 2 compares the lifestyle behaviors in subjects who did and did not develop CKD. The rates of healthy lifestyle behaviors of habitual moderate exercise (14.3% vs. 39.0%; p = 0.024), no late-night dinner (47.6% vs. 69.8%; p = 0.035), and no bedtime snacking (61.9% vs. 90.2%; p = 0.001) were significantly lower in the CKD group than in the non-CKD group. However, no significant differences were noted between these groups in the other lifestyle behaviors, including daily physical activity equal to walking, walking speed, eating speed, and skipping breakfast.

**Table 2:** The differences in the lifestyle behaviors in subjects with and without CKD.

	All (n = 316)	Developed CKD (n = 21)	Did not develop CKD (n = 295)	p value
Habitual moderate exercise: ≥30 min/session and ≥2 times/week, yes/no, n (%)	118 (37.3)/198 (62.7)	3 (14.3)/18 (85.7)	115 (39.0)/180 (61.0)	0.024
Physical activity equal to walking at least 1 h/day, yes/no, n (%)	124 (39.2)/192 (60.8)	5 (23.8)/16 (76.2)	119 (40.3)/176 (59.7)	0.133
Walking speed: compared with people of the same sex and age group, fast/slow, n (%)	193 (61.1)/123 (38.9)	10 (47.6)/11 (52.4)	183 (62.0)/112 (38.0)	0.191
Eating speed: compared with others, fast/slow, n (%)	122 (38.6)/194 (61.4)	11 (52.4)/10 (47.6)	111 (37.6)/184 (62.4)	0.180
Late-night dinners: ≥3 times/week, yes/no, n (%)	100 (31.6)/216 (68.4)	11 (52.4)/10 (47.6)	89 (30.2)/206 (69.8)	0.035
Bedtime snacking: ≥3 times/week, yes/no, n (%)	37 (11.7)/279 (88.3)	8 (38.1)/13 (61.9)	29 (9.8)/266 (90.2)	0.001
Skipping breakfast: ≥3 times/week, yes/no, n (%)	28 (8.9)/288 (91.1)	2 (9.5)/19 (90.5)	26 (8.8)/269 (91.2)	0.912
Smoking habit, yes/no, n (%)	68 (21.5)/248 (78.5)	3 (14.3)/18 (85.7)	65 (22.0)/230 (78.0)	0.404
Drinking habit, yes/no, n (%)	244 (77.2)/72 (22.8)	14 (66.7)/7 (33.3)	230 (78.0)/65 (22.0)	0.233

CKD, chronic kidney disease.

Table 3, Table 4 shows the differences in the changes in the coronary risk factors among the four categories of changes in lifestyle behaviors. No significant differences among these groups were noted in age, eGFR, and other coronary risk factors at baseline. After the follow-up, the eGFR level decreased in all groups. A significant interaction effect for group × time was observed in the eGFR level between maintaining a healthy lifestyle and maintaining a healthy lifestyle with respect to habitual moderate exercise, daily physical activity equal to walking, and bedtime snacking (all p < 0.05). Similarly, a significant interaction effect for group × time was observed in the waist circumference, systolic blood pressure (SBP), diastolic blood pressure (DBP), LDL-C, and HbA_1_c levels among all four lifestyle groups with respect to habitual moderate exercise, daily physical activity equal to walking, walking speed, and bedtime snacking (all p < 0.05).

**Table 3:** The influence of the changes in lifestyle behaviors on age, eGFR level, anthropometric indices, and blood pressure.

		Age at baseline, years	eGFR, mL/min/1.73 m^2^		BMI, kg/m^2^		Waist circumference, cm		SBP, mm Hg		DBP, mm Hg	
			Baseline	After	Baseline	After	Baseline	After	Baseline	After	Baseline	After
Habitual moderate exercise: ≥30 min/session and ≥2 times/week	Healthy–healthy	53.1 (6.3)	80.7 (6.0)	76.5 (6.2)*	23.2 (2.6)	23.1 (2.6)	82.6 (7.0)	82.9 (7.2)	127.5 (13.7)	131.6 (16.1)*	82.4 (10.0)	78.7 (12.0)*
	Unhealthy–healthy	51.4 (6.5)	81.9 (6.4)	76.8 (8.7)*	23.3 (3.3)	23.1 (3.3)	84.0 (9.1)	83.5 (9.1)	123.8 (15.1)	129.9 (19.6)*	80.6 (10.9)	78.9 (12.3)
	Healthy–unhealthy	51.7 (6.5)	80.9 (7.5)	76.5 (8.3)*	23.0 (2.8)	23.2 (2.9)	82.5 (7.7)	84.0 (8.3)	125.1 (13.8)	130.6 (18.3)*	82.0 (10.2)	79.0 (12.8)
	Unhealthy–unhealthy	52.3 (7.0)	81.3 (5.8)	74.7 (8.5)*	23.2 (2.6)	23.3 (2.8)	83.9 (7.1)	85.6 (7.6)*^,a,b,†^	126.7 (14.5)	132.0 (18.3)*	82.3 (9.8)	80.1 (13.2)

Physical activity equal to walking at least 1 h/day	Healthy–healthy	53.1 (7.0)	81.1 (6.3)	77.1 (6.8)*	23.0 (3.0)	23.0 (2.9)	82.6 (8.1)	83.2 (8.1)	128.2 (15.4)	133.4 (15.5)*	82.3 (10.0)	80.8 (12.1)
	Unhealthy–healthy	52.8 (6.4)	80.2 (6.0)	74.9 (8.0)*	23.5 (2.3)	23.2 (2.6)	84.1 (6.1)	84.0 (7.1)	125.5 (12.6)	127.0 (15.2)	81.7 (11.2)	78.2 (12.0)
	Healthy–unhealthy	52.0 (6.8)	81.0 (6.2)	76.0 (8.2)*	23.5 (2.8)	23.8 (3.1)	84.6 (7.4)	86.6 (8.8)*	126.9 (14.1)	131.9 (19.1)*	82.4 (8.4)	80.4 (11.9)
	Unhealthy–unhealthy	52.0 (6.6)	81.3 (6.1)	74.9 (8.5)*^,a,†^	23.4 (2.7)	23.4 (2.8)	83.3 (7.4)	84.7 (7.5)*	125.5 (14.2)	132.5 (19.3)*	82.3 (9.0)	79.6 (13.5)

Walking speed: compared with people of the same sex and age group	Healthy–healthy	52.2 (6.9)	81.1 (6.4)	76.0 (7.3)*	23.4 (2.7)	23.5 (2.8)	83.4 (7.4)	83.8 (7.0)	126.4 (13.4)	132.0 (17.5)*	81.7 (10.0)	79.6 (12.1)
	Unhealthy–healthy	52.1 (6.4)	81.6 (6.7)	76.0 (8.7)*	23.1 (3.1)	22.9 (2.9)	82.3 (8.2)	81.2 (8.0)	126.0 (17.2)	126.6 (17.2)	82.5 (9.9)	79.0 (12.2)*
	Healthy–unhealthy	53.2 (6.5)	80.7 (6.5)	75.0 (8.7)*	23.5 (2.8)	23.9 (3.0)*	84.0 (7.7)	86.6 (9.1)*^,a,b^	127.7 (15.0)	133.9 (18.5)*	83.2 (9.3)	79.9 (11.8)
	Unhealthy–unhealthy	52.6 (6.8)	81.4 (5.2)	75.1 (8.5)*	23.2 (2.6)	23.2 (2.7)	83.7 (7.4)	85.5 (8.1)*^,a,b,†^	125.8 (13.9)	131.9 (18.4)*	82.5 (9.7)	80.3 (14.5)

Eating speed: compared with others	Healthy–healthy	52.4 (7.3)	81.5 (6.3)	76.6 (7.5)*	23.3 (2.5)	23.5 (2.6)	83.3 (7.1)	84.3 (7.7)	125.7 (14.6)	130.9 (17.6)*	82.6 (9.4)	80.4 (11.6)
	Unhealthy–healthy	52.0 (5.9)	81.5 (5.6)	75.5 (8.1)*	23.7 (3.1)	23.4 (3.3)	84.4 (8.3)	84.3 (9.0)	125.5 (15.1)	130.2 (19.4)	81.4 (12.2)	78.7 (15.6)
	Healthy–unhealthy	52.8 (6.1)	81.9 (6.8)	75.0 (8.3)*	23.7 (2.3)	24.0 (2.7)	84.0 (5.3)	85.4 (6.4)	129.2 (11.6)	136.4 (13.8)*	82.1 (9.5)	80.6 (10.1)
	Unhealthy–unhealthy	52.9 (5.9)	80.1 (5.9)	74.6 (8.2)*	24.2 (2.3)	24.2 (3.5)	86.1 (6.4)	87.4 (6.5)	127.5 (13.1)	132.3 (17.9)*	83.8 (9.4)	82.4 (12.3)

Late-night dinners: ≥3 times/week	Healthy–healthy	52.4 (6.9)	81.3 (6.1)	76.3 (7.3)*	23.2 (2.6)	23.2 (2.8)	83.2 (6.9)	84.0 (7.4)	126.0 (14.0)	131.7 (18.4)*	82.2 (10.0)	79.0 (12.6)*
	Unhealthy–healthy	52.4 (6.1)	80.9 (6.4)	74.5 (8.7)*	23.8 (3.4)	24.0 (3.6)	84.4 (9.1)	85.6 (9.7)	129.3 (16.5)	135.8 (20.6)*	82.5 (10.1)	79.9 (14.6)
	Healthy–unhealthy	51.5 (6.1)	81.9 (7.6)	75.8 (8.8)*	23.0 (2.6)	23.1 (2.3)	82.6 (6.2)	84.5 (6.2)*	124.3 (12.2)	129.5 (15.5)*	83.4 (10.5)	80.9 (10.5)
	Unhealthy–unhealthy	51.9 (6.9)	81.8 (5.1)	75.3 (8.9)*	23.4 (2.7)	23.3 (2.5)	84.0 (8.2)	85.0 (8.1)	126.7 (14.6)	132.3 (14.5)*	82.9 (9.7)	81.2 (12.7)

Bedtime snacking: ≥3 times/week	Healthy–healthy	52.5 (6.8)	81.4 (6.2)	75.7 (7.0)*	23.3 (2.7)	23.2 (2.8)	83.8 (7.4)	84.3 (7.9)	82.9 (10.2)	79.1 (12.3)*	82.9 (10.2)	79.1 (12.3)*
	Unhealthy–healthy	51.0 (5.6)	80.1 (6.7)	75.3 (8.2)*	24.2 (2.7)	23.9 (2.1)	84.5 (6.8)	83.6 (5.2)	84.9 (11.4)	76.1 (9.4)*	84.9 (11.4)	76.1 (9.4)*
	Healthy–unhealthy	52.6 (6.9)	80.9 (4.5)	72.2 (8.9)*^,a^	23.3 (3.5)	23.6 (3.3)	83.5 (9.4)	85.8 (9.8)*^,a,b^	80.8 (9.2)	82.5 (18.5)^a,b^	80.8 (9.2)	82.5 (18.5)^a,b^
	Unhealthy–unhealthy	52.6 (6.8)	80.0 (6.3)	68.9 (8.3)*^,a,b,†^	23.5 (2.5)	23.7 (2.8)	83.9 (6.4)	86.1 (6.8)*^,a,b,†^	83.4 (6.8)	83.7 (11.0)^a,b,†^	83.4 (6.8)	83.7 (11.0)^a,b,†^

Skipping breakfast: ≥3 times/week	Healthy–healthy	52.5 (6.8)	81.0 (6.1)	75.6 (8.1)*	23.4 (2.7)	23.4 (2.9)	83.5 (7.3)	84.3 (7.9)	126.3 (14.4)	131.4 (18.0)*	82.8 (9.9)	79.5 (12.6)
	Unhealthy–healthy	51.9 (7.4)	80.9 (8.2)	74.4 (8.2)*	22.5 (2.7)	22.3 (2.6)	81.2 (7.8)	82.6 (7.3)	124.6 (14.2)	130.9 (21.7)*	82.9 (11.6)	79.4 (13.5)
	Healthy–unhealthy	52.1 (7.4)	82.7 (5.6)	76.9 (7.5)*	23.3 (3.6)	23.4 (2.9)	83.9 (9.2)	84.0 (8.3)	127.0 (14.1)	132.0 (12.4)*	84.9 (11.4)	81.4 (10.7)
	Unhealthy–unhealthy	52.6 (5.2)	82.4 (4.7)	75.5 (7.0)*	23.3 (1.9)	23.5 (2.5)	83.7 (5.0)	85.4 (6.5)	129.6 (11.1)	135.6 (17.4)*	82.3 (7.5)	79.8 (13.7)

Smoking habit	Healthy–healthy	52.4 (6.8)	81.9 (6.1)	75.8 (8.1)*	23.2 (2.7)	23.2 (2.8)	83.7 (7.5)	84.4 (8.0)	126.5 (14.4)	131.3 (17.6)*	82.4 (9.7)	78.6 (12.5)*
	Unhealthy–healthy	52.8 (7.5)	81.7 (4.9)	75.2 (6.9)*	23.3 (3.2)	23.7 (2.8)	84.7 (6.5)	85.2 (6.4)	126.2 (16.7)	131.7 (19.9)	80.2 (12.4)	78.1 (13.9)
	Unhealthy–unhealthy	52.4 (6.3)	82.0 (6.8)	77.2 (7.4)*	23.0 (2.6)	22.9 (2.7)	81.1 (7.1)	82.7 (7.3)	125.6 (12.4)	132.9 (18.5)*	82.1 (10.6)	80.0 (13.6)

Drinking habit	Healthy–healthy	52.9 (6.8)	81.1 (6.0)	75.5 (8.8)*	23.3 (2.8)	23.3 (3.1)	82.8 (7.7)	83.6 (8.6)	125.2 (13.7)	129.8 (17.7)*	81.2 (10.4)	78.6 (13.9)
	Unhealthy–healthy	49.9 (6.5)	82.2 (5.9)	75.6 (7.9)*	23.4 (2.1)	23.5 (2.4)	83.5 (4.8)	84.1 (5.6)	124.9 (12.5)	131.7 (17.0)*	82.3 (7.7)	81.6 (12.3)
	Healthy–unhealthy	50.9 (7.6)	80.1 (7.0)	76.1 (9.0)*	23.6 (3.1)	23.2 (3.2)	84.3 (7.5)	84.3 (9.6)	124.3 (16.3)	129.9 (21.3)*	78.7 (8.7)	76.1 (13.7)
	Unhealthy–unhealthy	52.2 (6.5)	81.3 (7.5)	76.0 (7.5)*	23.3 (2.8)	23.3 (2.8)	83.5 (7.8)	85.0 (7.9)*	125.6 (14.5)	130.4 (17.7)*	82.0 (10.4)	81.4 (12.3)

BMI, body mass index; eGFR, estimated-glomerular filtration rate; DBP, diastolic blood pressure; SBP, systolic blood pressure.

Data are expressed as mean (standard deviation).

*p < 0.05, compared to the values at baseline in each group.

^†^p < 0.05, group × time interaction.

^a^p < 0.05, compared to the healthy–healthy group.

^b^p < 0.05, compared to the unhealthy–healthy group.

**Table 4:** The influence of the changes in lifestyle behaviors on the blood lipid and glucose tolerance indices.

		LDL-C, mg/dL		HDL-C, mg/dL		Triglyceride, mg/dL		Fasting glucose, mg/dL		HbA_1_c, NGSP %	
		Baseline	After	Baseline	After	Baseline	After	Baseline	After	Baseline	After
Habitual moderate exercise: ≥30 min/session and ≥2 times/week	Healthy–healthy	116.3 (26.3)	116.9 (25.8)	60.9 (14.1)	62.5 (14.4)	104.9 (54.0)	104.6 (55.2)	101.6 (18.7)	100.8 (22.4)	5.6 (0.7)	5.5 (0.8)
	Unhealthy–healthy	120.6 (25.0)	115.8 (26.6)	55.3 (13.4)	56.9 (15.3)	126.4 (121.2)	124.6 (103.3)	97.1 (11.9)	97.0 (120.3)	5.6 (0.4)	5.4 (0.5)
	Healthy–unhealthy	118.6 (29.3)	115.9 (29.3)	58.7 (15.1)	59.8 (16.8)	113.3 (64.8)	123.5 (76.5)	96.1 (9.6)	99.8 (13.6)	5.4 (0.3)	5.6 (0.5)*
	Unhealthy–unhealthy	119.2 (27.5)	122.6 (26.8)	57.2 (12.8)	58.3 (14.4)	117.4 (76.0)	125.6 (80.5)	100.2 (17.0)	100.9 (19.0)	5.6 (0.6)	5.6 (0.7)

Physical activity equal to walking at least 1 h/day	Healthy–healthy	115.6 (28.5)	114.6 (24.4)	61.1 (13.5)	61.8 (14.7)	110.5 (102.5)	109.7 (83.8)	98.8 (14.3)	99.6 (19.5)	5.6 (0.7)	5.5 (0.8)
	Unhealthy–healthy	117.2 (26.5)	114.4 (23.5)	58.2 (12.6)	60.0 (14.4)	110.0 (57.0)	107.8 (55.2)	97.3 (15.0)	95.8 (17.1)	5.6 (0.5)	5.5 (0.6)
	Healthy–unhealthy	123.3 (26.4)	121.7 (28.0)	55.7 (12.0)	56.0 (13.2)	123.0 (95.4)	139.6 (84.1)	98.9 (12.7)	101.1 (18.6)	5.5 (0.4)	5.7 (0.6)*^,a^
	Unhealthy–unhealthy	119.3 (26.6)	123.3 (28.3)	57.1 (14.1)	59.0 (15.4)	116.4 (58.3)	123.4 (77.5)	101.6 (18.7)	102.2 (19.5)	5.6 (0.6)	5.7 (0.7)*^,a,b,†^

Walking speed: compared with people of the same sex and age group	Healthy–healthy	120.8 (30.6)	120.0 (28.8)	58.8 (13.9)	60.0 (15.3)	112.9 (63.5)	122.8 (81.4)	101.6 (19.5)	101.9 (20.9)	5.6 (0.7)	5.5 (0.7)
	Unhealthy–healthy	114.4 (21.6)	116.6 (24.4)	58.3 (13.4)	61.6 (15.6)	118.1 (86.2)	116.1 (77.2)	99.6 (11.1)	99.0 (20.4)	5.7 (0.4)	5.6 (0.9)
	Healthy–unhealthy	120.9 (24.9)	120.2 (29.5)	54.8 (10.9)	55.8 (12.7)	128.7 (114.4)	124.5 (89.4)	96.8 (11.1)	97.4 (12.7)	5.5 (0.3)	5.5 (0.6)
	Unhealthy–unhealthy	115.5 (23.2)	119.8 (23.0)	58.6 (14.3)	59.4 (14.7)	108.9 (69.5)	113.4 (64.0)	98.6 (14.6)	100.2 (19.6)	5.6 (0.6)	5.5 (0.6)

Eating speed: compared with others	Healthy–healthy	116.2 (24.6)	116.0 (25.6)	59.6 (14.4)	60.2 (15.8)	111.6 (64.2)	107.8 (68.9)	99.9 (17.7)	100.9 (19.1)	5.6 (0.7)	5.5 (0.8)
	Unhealthy–healthy	120.4 (27.3)	124.8 (29.5)	57.2 (12.1)	59.3 (12.8)	109.7 (51.2)	113.5 (71.3)	97.4 (10.6)	97.8 (16.5)	5.5 (0.3)	5.4 (0.5)
	Healthy–unhealthy	123.3 (27.8)	131.3 (26.5)	54.6 (12.6)	54.1 (10.7)	127.2 (125.8)	121.6 (106.3)	98.8 (15.3)	100.7 (20.9)	5.6 (0.5)	5.7 (0.7)
	Unhealthy–unhealthy	121.8 (29.7)	121.8 (27.0)	56.3 (12.5)	57.1 (14.1)	115.3 (59.7)	125.3 (78.8)	102.5 (15.7)	101.7 (21.7)	5.7 (0.5)	5.6 (0.6)

Late-night dinners: ≥3 times/week	Healthy–healthy	120.6 (28.4)	119.1 (25.1)	58.3 (13.4)	59.7 (14.9)	111.1 (81.3)	120.5 (83.2)	99.0 (18.3)	100.9 (21.6)	5.6 (0.6)	5.6 (0.7)
	Unhealthy–healthy	118.2 (25.4)	122.2 (34.0)	56.4 (14.3)	56.7 (15.3)	120.6 (60.7)	129.7 (65.4)	103.7 (17.0)	103.0 (21.9)	5.6 (0.5)	5.6 (0.7)
	Healthy–unhealthy	115.7 (27.7)	122.4 (28.1)	57.6 (13.8)	58.5 (14.0)	124.3 (66.7)	120.1 (76.2)	98.3 (13.1)	99.0 (15.6)	5.5 (0.5)	5.5 (0.7)
	Unhealthy–unhealthy	114.2 (23.3)	117.1 (26.0)	59.1 (13.6)	60.4 (15.1)	116.9 (80.4)	109.8 (69.2)	100.6 (10.0)	98.8 (11.0)	5.5 (0.5)	5.5 (0.6)

Bedtime snacking: ≥3 times/week	Healthy–healthy	118.7 (27.5)	117.8 (25.8)	58.7 (13.2)	60.1 (14.2)	109.1 (67.3)	107.5 (77.2)	99.6 (16.5)	100.2 (20.1)	5.6 (0.6)	5.5 (0.7)*
	Unhealthy–healthy	123.6 (26.9)	126.4 (26.8)	57.4 (13.5)	60.4 (16.1)	111.5 (56.0)	122.2 (62.7)	99.3 (10.9)	99.4 (16.0)	5.6 (0.4)	5.4 (0.5)*
	Healthy–unhealthy	119.3 (25.2)	131.8 (32.6)*^,a^	55.6 (13.0)	57.9 (17.3)	123.4 (89.3)	114.3 (65.0)	102.5 (16.6)	102.2 (15.3)	5.7 (0.7)	5.8 (0.8)^a,b^
	Unhealthy–unhealthy	114.2 (23.4)	128.7 (31.3)*^,a,†^	53.2 (17.8)	52.1 (16.3)	128.5 (123.3)	138.9 (105.8)	101.3 (19.1)	102.5 (20.5)	5.7 (0.5)	5.8 (0.8)^a,b,†^

Skipping breakfast: ≥3 times/week	Healthy–healthy	118.6 (27.2)	119.2 (27.0)	58.0 (13.5)	59.4 (14.9)	113.2 (78.2)	115.3 (73.4)	99.9 (16.7)	100.5 (20.5)	5.6 (0.6)	5.6 (0.7)
	Unhealthy–healthy	115.0 (24.4)	109.4 (23.5)	61.9 (16.2)	63.3 (16.3)	131.0 (87.3)	149.3 (125.7)	98.9 (16.8)	98.6 (11.9)	5.6 (0.7)	5.3 (0.6)
	Healthy–unhealthy	121.0 (28.5)	124.9 (25.0)	57.1 (13.4)	58.9 (15.5)	119.1 (54.8)	133.1 (64.4)	99.5 (16.1)	101.2 (18.0)	5.5 (0.3)	5.4 (0.4)*
	Unhealthy–unhealthy	119.2 (27.4)	133.1 (27.4)	57.2 (10.9)	57.3 (12.1)	120.0 (74.2)	132.6 (76.3)	102.3 (8.7)	98.9 (5.6)	5.5 (0.3)	5.5 (0.6)

Smoking habit	Healthy–healthy	119.4 (27.4)	121.5 (26.8)	58.2 (13.3)	59.3 (14.7)	120.7 (73.8)	127.7 (74.8)	100.2 (17.0)	100.3 (20.1)	5.6 (0.6)	5.5 (0.7)
	Unhealthy–healthy	114.5 (29.8)	108.5 (24.5)	54.6 (11.3)	56.5 (11.7)	115.7 (76.9)	115.7 (93.0)	100.7 (17.2)	101.5 (21.5)	5.7 (0.5)	5.6 (0.6)
	Unhealthy–unhealthy	115.7 (22.9)	114.0 (27.0)	59.6 (16.0)	61.1 (17.4)	129.3 (92.4)	124.3 (85.6)	98.8 (9.5)	102.3 (14.3)*	5.5 (0.4)	5.5 (0.6)

Drinking habit	Healthy–healthy	118.7 (23.9)	123.0 (27.7)	55.0 (12.8)	55.3 (13.3)	111.6 (84.2)	112.3 (60.9)	95.0 (11.7)	96.7 (19.5)	5.6 (0.4)	5.7 (0.8)
	Unhealthy–healthy	117.9 (34.2)	118.1 (30.7)	54.7 (12.6)	56.2 (13.3)	132.9 (121.9)	133.1 (103.1)	97.1 (10.5)	95.9 (11.2)	5.5 (0.4)	5.4 (0.5)
	Healthy–unhealthy	121.8 (32.5)	116.1 (24.6)	54.1 (12.9)	56.3 (14.9)	113.6 (63.7)	120.9 (85.8)	95.4 (13.3)	98.9 (21.7)	5.5 (0.4)	5.4 (0.5)
	Unhealthy–unhealthy	118.4 (24.5)	119.3 (25.9)	58.4 (13.5)	59.9 (15.0)	112.2 (64.2)	118.9 (75.2)	100.9 (10.0)	101.2 (20.5)	5.5 (0.6)	5.5 (0.7)

HDL-C, high-density lipoprotein cholesterol; HbA_1_c, hemoglobin A_1_c; LDL-C, low-density lipoprotein cholesterol; NGSP, national glycohemoglobin standardization program.

Data are expressed as mean (standard deviation).

*p < 0.05, compared to the values at baseline in each group.

^†^p < 0.05, group × time interaction.

^a^p < 0.05, compared to the healthy–healthy group.

^b^p < 0.05, compared to the unhealthy–healthy group.

Table 5 shows that the association between the change in eGFR level and the changes in other coronary risk factors from baseline to follow-up, determined using a simple regression analysis. The change in eGFR level negatively correlated with the changes in BMI (r = −0.120, p = 0.033), waist circumference (r = −0.121, p = 0.032), SBP (r = −0.261, p < 0.0001), DBP (r = −0.223, p < 0.0001), LDL-C (r = −0.155, p = 0.006), and HbA_1_c levels (r = −0.188, p = 0.0008). In a stepwise multiple regression analysis, the change in eGFR level was entered as a dependent variable, while the changes in BMI, waist circumference, SBP, DBP, LDL-C, and HbA_1_c levels were entered as independent variables. The change in eGFR level was independently associated with the changes in SBP and HbA_1_c level (r^2^ = 0.083, p < 0.0001) (Table 6).

**Table 5:** The association of the change in eGFR level and the changes in other coronary risk factors calculated using simple regression analysis.

	Coefficient of correlation	p value
Δ BMI	−0.120	0.033
Δ Waist circumference	−0.121	0.032
Δ SBP	−0.261	<0.0001
Δ DBP	−0.223	<0.0001
Δ LDL-C	−0.155	0.006
Δ HDL-C	0.031	0.583
Δ Triglyceride	−0.060	0.291
Δ Fasting glucose	−0.061	0.279
Δ HbA_1_c	−0.188	0.0008

BMI, body mass index; DBP, diastolic blood pressure; eGFR, estimated glomerular filtration rate; HbA_1_c, hemoglobin A_1_c; HDL-C, high-density lipoprotein cholesterol; LDL-C, low-density lipoprotein cholesterol; SBP, systolic blood pressure.

Data are expressed as the coefficient of correlation.

**Table 6:** The association of the change in eGFR level and the changes in other coronary risk factors determined using stepwise multiple regression analysis.

	β	r^2^	p value
Δ SBP	−0.223	0.083	<0.0001
Δ HbA_1_c	−0.136		

BMI, body mass index; DBP, diastolic blood pressure; eGFR, estimated-glomerular filtration rate; HbA_1_c, hemoglobin A_1_c; LDL-C, low-density lipoprotein cholesterol; SBP, systolic blood pressure.

Data are expressed as the coefficient of correlation. The change in eGFR was entered as a dependent variable. The following factors were entered as independent variables: the changes in BMI, waist circumference, SBP, DBP, LDL-C and HbA_1_c levels.

Table 7 shows the differences in the odds ratio (OR) for the incidence of CKD among the four categories of changes in lifestyle behaviors. In this multiple logistic regression analysis, the category of changes in lifestyle behaviors (healthy–healthy, unhealthy–healthy, healthy–unhealthy, and unhealthy–unhealthy) was a dependent variable, and the incidence of CKD was an independent variable. In the univariate analysis, maintaining an unhealthy lifestyle with respect to habitual moderate exercise, daily physical activity equal to walking, and late-night dinner resulted in a significantly higher OR for incidence of CKD than maintaining a healthy lifestyle behavior (OR 6.08; 95% confidence interval [CI], 1.14–13.60, OR 4.16; 95% CI, 1.13–11.95, and OR 5.89; 95% CI, 1.16–13.64, respectively). The change from a healthy to an unhealthy lifestyle and maintaining an unhealthy lifestyle with respect to bedtime snacking also resulted in a significantly higher ORs for incidence of CKD than maintaining a healthy lifestyle (OR 6.05; 95% CI, 2.45–12.27 and OR 11.02; 95% CI, 2.83–26.69, respectively).

**Table 7:** The influence of the changes in lifestyle behaviors on the incidence of CKD.

		Total	Developed CKD, n (%)	Univariate model		Multivariate model	
				Odds ratio (95% CI)	p value	Odds ratio (95% CI)	p value
Habitual moderate exercise: ≥30 min/session and ≥2 times/week	Healthy–healthy	83	1 (1.2)	1.00 (Ref.)	–	1.00 (Ref.)	–
	Unhealthy–healthy	39	2 (5.1)	3.78 (0.60–9.68)	0.414	2.83 (0.69–12.96)	0.310
	Healthy–unhealthy	34	3 (8.8)	4.48 (0.28–9.25)	0.397	4.13 (0.45–13.52)	0.289
	Unhealthy–unhealthy	160	15 (9.4)	6.08 (1.14–13.60)	0.027	8.94 (1.10–15.40)	0.040

Physical activity equal to walking at least 1 h/day	Healthy–healthy	82	2 (2.4)	1.00 (Ref.)	–	1.00 (Ref.)	–
	Unhealthy–healthy	46	3 (6.5)	1.97 (0.24–6.38)	0.532	2.29 (0.45–7.34)	0.271
	Healthy–unhealthy	42	3 (7.1)	2.19 (0.53–9.19)	0.179	2.07 (0.49–9.18)	0.229
	Unhealthy–unhealthy	146	13 (8.9)	4.16 (1.13–11.95)	0.032	2.91 (0.89–12.78)	0.078

Walking speed: compared with people of the same sex and age group	Healthy–healthy	148	5 (3.4)	1.00 (Ref.)	–	1.00 (Ref.)	–
	Unhealthy–healthy	41	3 (7.3)	1.47 (0.45–8.54)	0.298	1.17 (0.52–8.87)	0.279
	Healthy–unhealthy	45	5 (11.1)	3.69 (0.63–13.32)	0.190	3.57 (0.69–12.97)	0.109
	Unhealthy–unhealthy	82	8 (9.8)	2.48 (0.56–11.96)	0.263	3.09 (0.77–9.79)	0.114

Eating speed: compared with others	Healthy–healthy	174	7 (4.0)	1.00 (Ref.)	–	1.00 (Ref.)	–
	Unhealthy–healthy	46	3 (6.5)	2.46 (0.51–9.02)	0.264	1.66 (0.41–6.71)	0.474
	Healthy–unhealthy	20	3 (15.0)	3.37 (0.82–11.47)	0.117	3.20 (0.92–12.80)	0.094
	Unhealthy–unhealthy	76	8 (10.5)	2.74 (0.79–8.75)	0.231	2.81 (0.89–8.04)	0.097

Late-night dinners: ≥3 times/week	Healthy–healthy	182	7 (3.8)	1.00 (Ref.)	–	1.00 (Ref.)	–
	Unhealthy–healthy	43	3 (7.0)	1.57 (0.30–6.29)	0.591	1.88 (0.46–7.57)	0.377
	Healthy–unhealthy	33	3 (9.1)	2.17 (0.66–11.52)	0.236	2.50 (0.61–10.21)	0.202
	Unhealthy–unhealthy	58	8 (13.8)	5.89 (1.16–13.64)	0.006	4.00 (1.38–11.57)	0.011

Bedtime snacking: ≥3 times/week	Healthy–healthy	257	9 (3.5)	1.00 (Ref.)	–	1.00 (Ref.)	–
	Unhealthy–healthy	17	1 (5.9)	1.51 (0.21–5.40)	0.540	1.74 (0.21–9.63)	0.609
	Healthy–unhealthy	21	4 (19.0)	6.05 (2.45–12.27)	0.002	4.44 (1.05–13.96)	0.003
	Unhealthy–unhealthy	21	7 (33.3)	13.93 (2.97–23.89)	0.0001	11.02 (2.83–26.69)	0.0001

Skipping breakfast: ≥3 times/week	Healthy–healthy	266	18 (6.8)	1.00 (Ref.)	–	1.00 (Ref.)	–
	Unhealthy–healthy	17	1 (5.9)	0.17 (0.01–2.42)	0.190	0.86 (0.11–6.87)	0.888
	Healthy–unhealthy	21	1 (4.8)	0.63 (0.06–5.69)	0.705	0.69 (0.09–5.43)	0.724
	Unhealthy–unhealthy	12	1 (8.3)	3.13 (0.33–10.11)	0.323	1.25 (0.15–10.25)	0.833

Smoking habit	Healthy–healthy	250	18 (7.2)	1.00 (Ref.)	–	1.00 (Ref.)	–
	Unhealthy–healthy	25	0 (4.0)	0.60 (0.06–6.03)	0.660	0.54 (0.07–4.20)	0.554
	Unhealthy–unhealthy	41	3 (4.9)	0.77 (0.14–4.14)	0.764	0.66 (0.15–2.96)	0.588

Drinking habit	Healthy–healthy	52	6 (11.5)	1.00 (Ref.)	–	1.00 (Ref.)	–
	Unhealthy–healthy	40	3 (7.5)	0.99 (0.17–5.84)	0.994	0.62 (0.15–2.66)	0.521
	Healthy–unhealthy	18	1 (5.6)	0.33 (0.03–4.10)	0.388	0.45 (0.05–4.03)	0.476
	Unhealthy–unhealthy	206	11 (5.3)	0.51 (0.15–1.78)	0.290	0.43 (0.15–1.23)	0.116

BMI, body mass index; CI, confidence interval; CKD, chronic kidney disease; DBP, diastolic blood pressure; eGFR, estimated-glomerular filtration rate; HbA_1_c, hemoglobin A_1_c; LDL-C, low-density lipoprotein cholesterol; SBP, systolic blood pressure.

In this analysis, the changes in lifestyle behaviors were dependent variables, and the incidence of CKD was an independent variable. In the multivariate model, age, BMI, waist circumference, SBP, DBP, LDL-C, HbA_1_c, and eGFR levels at baseline were entered as adjusted factors.

In multivariate analysis, age, BMI, waist circumference, SBP, DBP, LDL-C, HbA_1_c, and eGFR levels at baseline were entered as adjusted factors. After adjusting for these factors, maintaining an unhealthy lifestyle with respect to habitual moderate exercise and late-night dinner resulted in a significantly higher OR for incidence of CKD than maintaining a healthy lifestyle behavior (OR 8.94; 95% CI, 1.10–15.40 and OR 4.00; 95% CI, 1.38–11.57, respectively). In addition, changing from a healthy to an unhealthy lifestyle and maintaining an unhealthy lifestyle with respect to bedtime snacking also resulted in a significantly higher ORs for incidence of CKD than maintaining a healthy lifestyle (OR 4.44; 95% CI, 1.05–13.96 and OR 11.02; 95% CI, 2.83–26.69, respectively).

## Discussion

The major finding of the present study was that maintaining an unhealthy lifestyle with respect to habitual moderate exercise and late-night dinner significantly increased the incidence of CKD versus maintaining a healthy lifestyle. In addition, changing from a healthy to an unhealthy lifestyle and maintaining an unhealthy lifestyle with respect to bedtime snacking also significantly increased the incidence of CKD versus maintaining a healthy lifestyle. Furthermore, a stepwise multiple regression analysis demonstrated that the change in eGFR level was dependently associated with changes in SBP and HbA_1_c level. It is well known that elevations of blood pressure and blood glucose levels are the important independent risk factors for development of renal dysfunction.^24^, ^25^, ^26^ Moreover, the number of patients with ESRD in Japan is continuously increasing according to an increase in the type 2 diabetes.^1^ Thus, our current findings suggest that an increasing in SBP and HbA_1_c level were associated with the reduction in renal function, and we consider that early lifestyle intervention is required, especially focusing on blood pressure and glycemic controls for preventing the development of CKD.

Adherence to a healthy lifestyle is well known to be related to a decreased incidence of metabolic syndrome, type 2 diabetes, hypertension, and dyslipidemia.^27^, ^28^, ^29^, ^30^ We think that a similar mechanism underlies the relationship between healthy lifestyle behaviors and reduced risk of CKD, and maintaining a healthy lifestyle or improving an unhealthy one therefore plays an important role in the preventing these diseases. However, despite our promising findings, the association between any changes in lifestyle behaviors and the prevention of CKD remains unclear at present. Ricardo et al^31^ evaluated the influence of four health lifestyle factors (regular physical activity, maintaining a reasonable body weight, not smoking, and enjoying a healthy eating style) on the incidence of CKD progression, atherosclerotic events, and all-cause mortality. Those authors showed that the incidence of CKD, atherosclerotic events, and all-cause mortality was significantly reduced with increasing healthy lifestyle scores. Our recent cross-sectional and longitudinal studies^13^, ^14^ also found that the accumulation of healthy lifestyle behaviors, especially those related to habitual moderate exercise and no bedtime snacking, may be predictive factors for the incidence of CKD. In our results, the incidence of CKD was reduced among subjects who maintained a healthy lifestyle or improved an unhealthy one compared to maintaining an unhealthy lifestyle, findings which are consistent with those of prior research. Thus, our current findings support the possibility that maintaining a healthy lifestyle or improving an unhealthy one is important to prevent the development of CKD, ESRD, and CVD.

In the present study, maintaining a healthy lifestyle or improving an unhealthy one with respect to habitual moderate exercise, late-night dinner, and bedtime snacking was correlated with the incidence of CKD. However, no significant associations were noted between the changes in other lifestyle behaviors and the development of CKD. A few studies recently demonstrated that individual lifestyle behaviors relating to exercise, physical activity, and eating styles related to the development of renal dysfunction. For example, Robinson-Cohen et al^32^ reported that each 60-min increase in weekly physical activity was associated with an annual eGFR reduction of 0.5% per year. Similarly, Bharakhada et al^33^ demonstrated that high levels of physical activity and low levels of sitting time are related to a lower prevalence of CKD, independent of other risk factors. A previous meta-analysis^10^ showed that aerobic exercise intervention can improve aerobic capacity, muscular function, cardiovascular function, walking capacity, and health-related quality of life in patients with CKD and dialysis. Furthermore, unhealthy eating styles, such as high intake of dietary animal fat, sodium, and soft drinks has been reported to lead to reduced renal function.^34^, ^35^, ^36^ The Framingham Heart Study^37^ examined the association between lifestyle characteristics and the incidence of renal dysfunction and found that better diet quality is associated with a decreased risk of incidence of renal dysfunction. Additionally, Mekary et al^38^ demonstrated the consuming snacks beyond the three main meals (breakfast, lunch, and dinner) was related to an elevated risk of developing type 2 diabetes. These studies suggest that maintaining a healthy lifestyle or improving an unhealthy one with respect to habitual moderate exercise and healthy eating style may be important to prevent the incidence of CKD.

Several prior studies have reported that lack of habitual moderate exercise, eating late-night dinner, and bedtime snacking were associated with mortality and the incidence/prevalence of CVD, metabolic syndrome, type 2 diabetes, hypertension, and dyslipidemia.^36^, ^39^ Our results demonstrated that the lack of habitual moderate exercise, eating late-night dinner, and bedtime snacking may increase the risk of CKD, as well as the risk of introduction of dialysis in middle-aged and older men. Therefore, we believe that the evaluation of healthy lifestyle behaviors, with a particular focus on regular exercise habit and healthy eating styles, such as avoiding late-night dinner and bedtime snacking, are important when practicing lifestyle intervention to prevent renal dysfunction.

### Study limitations and clinical implications

There are several limitations in this study. First, the limited study population resulted in a small number of subjects, who were predominantly middle-aged and older men and did not have any health complications. Therefore, there is potential selection bias in this study, as study subjects may have included more CKD subjects with slowly declining renal function than CKD subjects with rapid deterioration. As such, whether or not our results are generalizable to women, patients with ESRD, or subjects with other complications remains unclear. Second, unfortunately, in this study, the causality of proteinuria or microalbuminuria and the changes in other cardiovascular risk factors could not be clarified because the number of subjects with proteinuria was small. Finally, we assessed the eGFR as calculated using the CKD-EPI equations (modified by a Japanese coefficient^17^) and proteinuria as markers of the renal function. To fully clarify the influence of changes in lifestyle behaviors on the renal dysfunction, other markers of renal function, such as urinary protein excretion, microalbuminuria, or cystatin C, should be concurrently evaluated. However, we were unable to measure any additional indices of renal function because current research was evaluated within the constraints of the health check-up.

However, despite these limitations, the present study is the first to elucidate the relationship with the changes in lifestyle behaviors and the incidence of CKD in a long-term follow-up study. We believe that our results support the notion that maintaining a healthy lifestyle or improving an unhealthy one can prevent the incidence of CKD. Furthermore, although the serum Cr level can be easily measured as part of a routine clinical evaluation, the eGFR is a strong predictive factor of future incidence of CVD and ESRD and is more useful for this purpose than the serum Cr level.^19^, ^20^ On the basis of recent several studies, it has been observed that the development of CKD is also closely related to unhealthy lifestyles behaviors, such as smoking, heavy alcohol intake, obesity, lack of exercise habit, and an unhealthy eating styles.^7^, ^8^, ^9^, ^10^, ^11^, ^12^ Therefore, our results suggest a clear link between maintaining a healthy lifestyle or improving an unhealthy one and the development of CKD and support the hypothesis that maintaining a healthy lifestyle or improving an unhealthy one helps to prevent the development of CVD and ESRD. Given the present findings, we consider that lifestyle intervention should be provided, particularly with a focus on regular exercise habit and healthy eating styles, such as avoiding no late-night dinner and no bedtime snacking, to reduce the development of CKD. Further investigation in a larger population, including subjects with other complications, is required to more precisely clarify the mechanisms underlying this association and the clinical implications of long-term intervention.

## Conclusions

This retrospective study assessed the relationship between changes in lifestyle behaviors and the incidence of CKD in middle-aged and older men. We observed that maintaining an unhealthy lifestyle with respect to habitual moderate exercise, late-night dinner, and bedtime snacking resulted in significantly higher OR of incident CKD compared to maintaining a healthy lifestyle. These results suggest that the lack of habitual moderate exercise, eating late-night dinner, and bedtime snacking may increase the risk of CKD. Therefore, it is important for middle-aged and older Japanese men to engage in habitual moderate exercise and to avoid late-night dinner or bedtime snacking, so that they may reduce the risk of dialysis therapy.

## Conflicts of interest

None declared.
